# Retinal Detachment in Down Syndrome: Characteristics and Surgical Outcomes

**DOI:** 10.1155/2016/6971591

**Published:** 2016-03-30

**Authors:** Badr O. AlAhmadi, Sulaiman M. Alsulaiman, J. Fernando Arevalo

**Affiliations:** ^1^King Khalid Eye Specialist Hospital, P.O. Box 7191, Riyadh 11462, Saudi Arabia; ^2^The Wilmer Eye Institute, Johns Hopkins University School of Medicine, 600 N. Wolfe Street, Maumenee 708, Baltimore, MD 21287, USA

## Abstract

*Purpose.* To determine the functional and anatomic outcomes of rhegmatogenous retinal detachment (RRD) surgery in patients with Down syndrome.* Methods.* A retrospective chart review was performed of patients with Down syndrome who had undergone surgery for RRD at King Khalid Eye Specialist Hospital between 1995 and 2014.* Results.* A total of 245 patients with Down syndrome were evaluated during the study period. Eighteen eyes of 15 patients (6.1%) with RRD were identified. Three out of 15 patients (20%) presented with bilateral retinal detachment. All eyes presented with macula off retinal detachment. The retina was successfully reattached in 16/18 (88.8%) eyes after a mean follow-up of 48 months. The final postoperative visual acuity ranged from light perception to 20/125 (median: hand motion) (11/18 eyes).* Conclusions.* The anatomic success rate of retinal reattachment surgery in patients with Down syndrome is comparable to the general population. Patients with Down syndrome should undergo regular ophthalmic examinations for early diagnosis. Despite late diagnosis and the presence of proliferative vitreoretinopathy (PVR) in some patients, favorable anatomical outcomes can be achieved.

## 1. Introduction

Down syndrome (DS) is one of the most prevalent genetic disorders caused by trisomy of all or part of chromosome 21 [1]. It is associated with significant mental disability and delays in physical growth with characteristic facial features. DS is the most common chromosome abnormality in humans [2], occurring in about 1 per 1000 babies born yearly [3]. It is more common in males than females [4, 5].

Several studies have shown that various ocular disorders are commonly seen in DS [6–8]. The prevalence of refractive errors (hyperopia, myopia, and astigmatism) ranged between 3% and 62.3% [6, 8] and strabismus between 5% and 57% [6, 8–10], with higher prevalence of esotropia than exotropia in all the studies [6, 8–10]. Other ocular manifestations include reduced amplitude of accommodation (26%–91.8%) [8], eyelid abnormalities (1%–6.7%) [9, 10], blepharitis (30%–47%) [7, 11], Brushfield spots (36%–81%) [7, 11], and glaucoma (0.8%–6.7%) [6, 10]. Retinal disorders such as retinoblastoma (1.7%) [12] and RRD were also reported (1.7%) [12]. There is limited data available in the literature regarding the incidence of RRD and outcomes of retinal surgery in Down syndrome [12, 13].

The objective of this study was to report the characteristics and surgical outcomes of RRD associated with Down syndrome.

## 2. Methods

The Internal Review Board/Ethics Committee at King Khaled Eye Specialist Hospital (KKESH) approved this study. A retrospective chart review was performed of all patients with RRD associated with Down syndrome from 1995 to 2014. The charts were reviewed to obtain the following data: age of the patient, laterality, duration of complaint, date of presentation, preoperative best-corrected visual acuity (BCVA), lens status on presentation, attachment or detachment of macula, extent of detachment, number, type, and location of retinal breaks, presence and grade of proliferative vitreoretinopathy (PVR), date of surgery, type of surgery performed, postoperative visits including examination under anesthesia (EUA) or examination under sedation (EUS), postoperative BCVA, postoperative lens status and anatomic outcome of the retina, subsequent surgery, anatomical and visual outcome at the last follow-up, duration of follow-up, and number of EUAs and EUSs.

Surgical techniques utilized included a standard 3-port 20/23-gauge pars plana vitrectomy (PPV) starting with a core vitrectomy followed by induction of a posterior vitreous detachment (PVD) along with shaving of the vitreous base. Endolaser photocoagulation was applied around the retinal breaks with or without 360-degree laser photocoagulation under air or perfluorocarbon liquid (PFCL). The intraocular tamponade of nonexpansile concentration of gas [sulphur hexafluoride (SF6), perfluoropropane (C3F8)] or silicone oil (5000 centistokes or heavy silicone oil) was selected according to the surgeon's discretion. In cases that were combined with a scleral buckle (SB), a 360-degree encircling band (# 41, # 42, or # 240) was used, and in 3 cases a segmental silicone tire (# 276, # 277, and # 287) was placed under the encircling band. If a scleral buckling was utilized as the sole treatment, the breaks were localized, cryopexy was performed around the breaks, and 360-degree encircling band was used with or without tires.

## 3. Results

A total of 245 Down syndrome patients were evaluated during the study period. Eighteen eyes of 15 patients (6.1%) with RRD were identified. There were 11 males and 4 females, with a mean age of 11.3 years (range, 4 to 26 years). The left eye was involved in 10 (55.5%) patients while the right eye was involved in 8 (44.5%) patients. Three out of 15 patients (20%) presented with bilateral retinal detachment. Verified or suspected ocular or head trauma was implicated in 5 (27.7%) of the 18 eyes, no trauma in 4 (22.2%) eyes, and unknown history of trauma in 9 (50%) eyes. At presentation, 2 (11.1%) eyes had a clear lens, 9 (50%) eyes had a cataractous lens, 4 (22.2%) eyes were pseudophakic, and 3 (16.6%) eyes were aphakic. All eyes presented with macula off retinal detachment. The detachment was total in 15 (83.3%) eyes, 3 quadrants of involvement in 2 (11.1%) eyes, and 2 quadrants of involvement in one (5.5%) eye. Two (11.1%) eyes presented with PVR grade B and 6 (33.3%) eyes with grade C (2 anterior, 1 posterior, 2 anterior and posterior locations, and 1 with no location documented). Eight eyes presented with 1 or more horseshoe tears (HST), 3 eyes with giant retinal tear (GRT), 6 eyes with atrophic or operculated holes, and 1 eye with retinal dialysis and in 1 eye, the break was unidentifiable (Table 1).

In the relatively cooperative patients in whom visual acuity could be assessed, preoperative visual acuity ranged from following light to counting fingers (median: following and fixation) (10/18 eyes) and final postoperative best-corrected visual acuity ranged from light perception to 20/125 (median: hand motion) (11/18 eyes). An encircling scleral band alone was performed in 6 (33.3%) eyes. Pars plana vitrectomy alone was performed in one (5.5%) eye. Combined buckle-vitrectomy was performed in 11 (61.1%) eyes. A segmental solid tire under the break was used in addition to the buckle in 5 (27.7%) eyes. Pars plana lensectomy (PPL) was performed in 5 (27.8%) eyes that were kept aphakic except one patient who underwent secondary intraocular lens (IOL) implantation. One eye underwent extracapsular cataract extraction during the primary procedure due to dense cataract. One eye underwent lens aspiration through anterior approach. In two eyes, the cataract was not significant enough to warrant removal. Three (16.6%) patients underwent EUS in the first postoperative visit, 1 (5.5%) patient underwent EUS in the second postoperative visit, and none had EUS in the last visit. No patients needed EUA.

Common complications noted after surgery included cataract formation in 1 (5.5%) eye, posterior capsular opacity in 1 (5.5%) eye, high intraocular pressure in 3 (16.6%) eyes, silicone oil emulsification in 1 (5.5%) eye, and endophthalmitis in 1 (5.5%) eye. Persistent or recurrent retinal detachment associated with PVR was noted in 6 (33.3%) eyes. All of the 6 eyes (2 with persistent detachment and 4 with recurrent detachment) underwent repeated surgery and successful reattachment was achieved in 4 of them. Because of the small number of eyes, a meaningful statistical analysis to assess the effect of the extent of detachment, number and type of breaks, and preexisting PVR on final outcome could not be performed.

Follow-up ranged from 3.1 to 210.5 months (mean, 48.0 months). The retina was successfully reattached after one procedure in 12/18 (66.6%) eyes and after a second procedure in 4/18 (22.2%) eyes. Therefore, the final success rate was 88.8% (16/18 eyes). The results are summarized in Table 2.

## 4. Discussion

We report the results of 18 eyes of Down syndrome patients who had retinal detachment repair. The mean age at presentation in our series is 11.3 years compared to 13 years in the series reported by Ahmad and Pruett [13]. The male predominance in our series may be related to the fact that Down syndrome affects males more frequently or to the possibility of trauma. In the report of 6 patients with DS and RRD by Ahmad and Pruett, trauma was implicated in 55% of cases [13]. Sarrazin et al. [14] found that more than half cases with traumatic retinal detachment presented late, probably because of its late development. The late development of traumatic retinal detachment in young patients may be due to the tight adherence between the retina and vitreous gel and the absence of posterior vitreous detachment (PVD) and vitreous liquefaction [14]. In our study, the onset of detachment is difficult to ascertain; however, 8 (44.4%) eyes had some degree of PVR and signs of chronicity.

In the literature, the anatomical success rate for retinal detachment repair varies depending on the surgical approach. Pars plana vitrectomy and silicone oil, PPV and gas with or without a combined scleral buckling, demonstrated success rates between 73% and 95% [15, 16]. For 20-gauge surgery, success ranges from 74.3% to 89.3% and for 23-gauge surgery, success ranges from 80% to 91.7% [17, 18]. For buckle surgery, surgical success ranges between 76% and 91% [19, 20] and, for cases with PVR, between 68% and 84% [21, 22]. In our study, the anatomical success rate was 88.89%, which was comparable to rates in the literature.

DS is the most common known cause of mental retardation and is characterized by developmental delay and language and cognitive abnormalities. Patients with DS are more likely to present with chronic and complex retinal detachment that is more difficult to repair [13]. Additionally, there is greater likelihood of requiring a combined procedure and silicone oil tamponade. In our study, silicone oil was used in 10 (55.56%) eyes and this may be an advantage because of noncompliance with positioning postoperatively in this group of patients. However, complications associated with silicone oil should be considered including cataract, glaucoma, corneal decompensation, and the need for a second surgery for silicone oil removal [23].

Patients with Down syndrome are usually uncooperative during examination and instillation of medications. In addition, the operated eye is at risk of complications due to rubbing or inadvertent contusion [24]. Therefore, in some patients, frequent examination under sedation or anesthesia for full assessment may be needed. Poor prognosis may be related to the late discovery of detachment and the difficulty in compliance with postoperative instructions.

The American Academy of Pediatrics recommended the following for health supervision for Children with Down syndrome: red reflex evaluation at birth, ophthalmologic examination by a pediatric ophthalmologist within 6 months, and annual ophthalmologic examination until 5 years of age, every two years until 13 years of age, and then every 3 years thereafter unless otherwise indicated [25]. We believe that more frequent ophthalmologic examination (at least annually) may be required to avoid late recognition of such devastating complication.

Limitations of our study include the small sample size and the retrospective, descriptive nature of our study without a control group. In addition, the incidence of RRD in Down syndrome in our population may be bias as we are a tertiary referral center. Furthermore, several surgeons with a variety of surgical techniques performed our cases.

In summary, the anatomic success rate of retinal reattachment surgery in patients with Down syndrome is comparable to the general population. These patients should undergo periodic ophthalmic examination to avoid delayed diagnosis. Despite late diagnosis and the presence of PVR in some patients, favorable anatomical outcomes can be achieved.

## Figures and Tables

**Table 1:** Characteristics and surgical outcomes in fifteen patients with retinal detachment in Down syndrome.

Case	Age	Eye	Lens	Number of retinal breaks	Type of retinal breaks	Macula	Quadrant of retinal detachment	PVR grade	Surgery	First subsequent surgery	Second subsequent surgery	Buckle details	Anatomical results	Follow-up
1	23	OS	Clear	7	HST	Off	Total	Not documented	PPV/SB/ELX/SO	ELX/Phaco + PC IOL/SO removal		Band	Flat	5.97
2	12	OS	Pseudophakia	1	HST	Off	Total	C-anterior + posterior	PPV/SB/ELX/SO	SO removal		Band	Flat	5.5
3	4	OD	Aphakia	1	HST	Off	Total	C-anterior	SB/CRYO	PPV/ELX/C3F8		Band	Flat	39.67
4	4	OS	Cataract	1	GRT	Off	Total	Not documented	PPV/SB/PPL/RR/ELX/SO			Band	Flat	3.63
5	11	OD	Cataract	1	GRT	Off	Total	C	PPV/SB/PPL/RR/ELX/SO			Band	Flat	13.93
6	17	OS	Cataract	2	HST + dialysis	Off	Total	Not documented	PPV/PPL/ELX/C3F8	PPV/SB/ELX/SO	Secondary IOL and SO removal	n/a	Flat	8.3
7	8	OD	Aphakia	n/a	n/a	Off	Total	C-anterior + posterior	PPV/SB/RR/ELX/choroidal drainage/SO			Band and tire	Detached	8.6
8	13	OD	Clear	2	Operculated hole	Off	Three	Not documented	SB/CRYO	PPV/ELX/CRYO/C3F8	SB removal and intravitreal AB	Band and tire	Flat	12.37
9	6	OS	Cataract	1	Operculated hole	Off	Total	C-anterior	PPV/SB/PPL/RR/ELX/C3F8			Band and tire	Flat	163.27
10	5	OS	Pseudophakia	1	HST	Off	Total	C-posterior	PPV/SB/ELX/heavy SO			Band	Flat	3.83
11	5	OS	Aphakia	3	HST + HST + operculated hole	Off	Total	B	PPV/SB/ELX/SO			Band	Detached	144
12	26	OD	Cataract	2	HST	Off	Total	Not documented	SB/CRYO/ECCE	PPV/RR/ELX/C3F8		Band	Flat	15.73
13	13	OU	Cataract	2	Operculated hole	Off	Three	Not documented	SB/CRYO			Band	Flat	3.23
13		OU	Cataract	2	HST	Off	Two	Not documented	SB/CRYO			Band and tire	Flat	3.1
14	8	OU	Cataract	1	GRT	Off	Total	Not documented	PPV/SB/PPL/ELX/SO			Band and tire	Flat	210.5
14		OU	Cataract	2	HST	Off	Total	B	SB/lens aspiration/CRYO			Band	Flat	203.97
15	14	OU	Pseudophakia	1	Operculated hole	Off	Total	Not documented	PPV/SB/ELX/SO				Flat	10.53
15		OU	Pseudophakia	1	Operculated hole	Off	Total	Not documented	PPV/SB/ELX/C3F8				Flat	8.6

OD: right eye; OS: left eye; OU: both eyes; HST: horseshoe tear; GRT: giant retinal tear; PPV: pars plana vitrectomy; SB: scleral buckle; PPL: pars plana lensectomy; RR: relaxing retinectomy; ELX: endolaser; CRYO: cryotherapy; SO: silicon oil; C3F8: perfluoropropane; ECCE: extracapsular cataract extraction; PC IOL: posterior chamber intraocular lens; IOL: intraocular lens; AB: antibiotic; PVR: proliferative vitreoretinopathy; n/a: not available.

**Table 2:** Summary of the result.

Number of eyes	18				
Mean age	11.3 years (range, 4 to 26 years)				
Eye					
Right	8				
Left	10				
Both	3				
Lens status					
Clear	2 (11.1%)				
Cataractous	9 (50%)				
Pseudophakia	4 (22.2%)				
Aphakia	3 (16.6%)				
Type of retinal breaks	8 eyes with HST	3 eyes with GRT	8 eyes with atrophic or operculated holes	1 eye with retinal dialysis	1 eye with unidentifiable break
Macula	All eyes with macula off detachment				
Quadrant of retinal detachment	Total in 15 (83.3%) eyes	3 quadrants in 2 (11.1%) eyes	2 quadrants in one (5.5%) eye		
PVR grade					
Grade B	2 (11.1%)				
Grade C	6 (33.3%)				
Anterior	2				
Posterior	1				
Anterior and posterior	2				
Undetermined	1				
Surgery	SB in 6 (33.3%) eyes	PPV in 1 (5.5%) eye	PPV + SB in 11 (61.1%) eyes		
PPL	In 5 (27.8%) eyes				
First subsequent surgery	PPV in 3 (16.7%) eyes	PPV + SB in 1 (5.5%) eye	SO removal in 1 (5.5%) eyes	Phaco + PC IOL/SO removal in 1 (5.5%) eye	
Second subsequent surgery	Secondary IOL and SO removal in 1 (5.5%) eye	SB removal and intravitreal AB in 1 (5.5%) eye			
Buckle details	Band + tire in 5 (27.7%) eyes	Band in 12 (66.7%) eyes			
Preoperative BCVA	From following light to counting fingers (median: following and fixation) (10/18 eyes)				
Postoperative BCVA	From light perception to 20/125 (median: hand motion) (11/18 eyes)				
Anatomical results	Flat in 88.8% (16 eyes)	Detached in 11.2% (2 eyes)			
Follow-up	Ranged from 3.1 to 210.5 months (mean, 48.0 months)				

HST: horseshoe tear; GRT: giant retinal tear; PPV: pars plana vitrectomy; SB: scleral buckle; PPL: pars plana lensectomy; PC IOL: posterior chamber intraocular lens; IOL: intraocular lens; AB: antibiotic; PVR: proliferative vitreoretinopathy.

## Additional Points

Down syndrome patients present with chronic and complex rhegmatogenous retinal detachment that is more difficult to repair. Periodic ophthalmic examination is required for Down syndrome patients for timely diagnosis. The retinal reattachment rate in patients with Down syndrome is comparable to the general population.

## References

[1] Patterson D. (2009). Molecular genetic analysis of Down syndrome. *Human Genetics*.

[2] Malt E. A., Dahl R. C., Haugsand T. M. (2013). Helse og sykdom hos voksne med downs syndrom. *Tidsskrift for den Norske Laegeforening*.

[3] Weijerman M. E., de Winter J. P. (2010). Clinical practice: the care of children with Down syndrome. *European Journal of Pediatrics*.

[4] Bell J. A. (1991). The epidemiology of Down's syndrome. *Medical Journal of Australia*.

[5] Bishop J., Huether C. A., Torfs C., Lorey F., Deddens J. (1997). Epidemiologic study of Down syndrome in a racially diverse California population. *American Journal of Epidemiology*.

[6] Kim J. H., Hwang J.-M., Kim H. J., Yu Y. S. (2002). Characteristic ocular findings in Asian children with Down syndrome. *Eye*.

[7] Shapiro M. B., France T. D. (1985). The ocular features of Down's syndrome. *American Journal of Ophthalmology*.

[8] Ebeigbe J. A., Akpalaba R. (2006). Ocular health status of subjects with Down's syndrome in Benin City, Nigeria. *African Journal of Medicine and Medical Sciences*.

[9] Motley W. W., Saltarelli D. P. (2011). Ophthalmic manifestations of mosaic Down syndrome. *Journal of AAPOS*.

[10] Caputo A. R., Rudolph S. W., Reynolds D. R. (1989). Down syndrome: clinical review of ocular features. *Clinical Pediatrics*.

[11] Berk A. T., Saatci A. O., Erçal M. D., Tunç M., Ergin M. (1996). Ocular findings in 55 patients with Down's syndrome. *Ophthalmic Genetics*.

[12] Liza-Sharmini A. T., Azlan Z. N., Zilfalil B. A. (2006). Ocular findings in Malaysian children with Down syndrome. *Singapore Medical Journal*.

[13] Ahmad A., Pruett R. C. (1976). The fundus in mongolism. *Archives of Ophthalmology*.

[14] Sarrazin L., Averbukh E., Halpert M., Hemo I., Rumelt S. (2004). Traumatic pediatric retinal detachment: a comparison between open and closed globe injuries. *American Journal of Ophthalmology*.

[15] Tanner V., Minihan M., Williamson T. H. (2001). Management of inferior retinal breaks during pars plana vitrectomy for retinal detachment. *British Journal of Ophthalmology*.

[16] Martínez-Castillo V., Verdugo A., Boixadera A., García-Arumí J., Corcóstegui B. (2005). Management of inferior breaks in pseudophakic rhegmatogenous retinal detachment with pars plana vitrectomy and air. *Archives of Ophthalmology*.

[17] Albrieux M., Rouberol F., Bernheim D., Romanet J.-P., Chiquet C. (2011). Comparative study of 23-gauge vitrectomy versus 20-gauge vitrectomy for the treatment of rhegmatogenous retinal detachment. *Graefe's Archive for Clinical and Experimental Ophthalmology*.

[18] Lewis S. A., Miller D. M., Riemann C. D., Foster R. E., Petersen M. R. (2011). Comparison of 20-, 23-, and 25-gauge pars plana vitrectomy in pseudophakic rhegmatogenous retinal detachment repair. *Ophthalmic Surgery Lasers and Imaging*.

[19] Pastor Jimeno J. C., Fernández I., De La Rodríguez Rúa E. (2008). Surgical outcomes for primary rhegmatogenous retinal detachments in phakic and pseudophakic patients: the Retina 1 project-report 2. *British Journal of Ophthalmology*.

[20] Heimann H., Bartz-Schmidt K. U., Bornfeld N. (2007). Scleral buckling versus primary vitrectomy in rhegmatogenous retinal detachment: a prospective randomizedmulticenter clinical study. *Ophthalmology*.

[21] Han D. P., Rychwalski P. J., Mieler W. F., Abrams G. W. (1994). Management of complex retinal detachment with combined relaxing retinotomy and intravitreal perfluoro-n-octane injection. *American Journal of Ophthalmology*.

[22] Fisher Y. L., Shakin J. L., Slakter J. S., Sorenson J. A., Shafer D. M. (1988). Perfluoropropane gas, modified panretinal photocoagulation, and vitrectomy in the management of severe proliferative vitreoretinopathy. *Archives of Ophthalmology*.

[23] Ferrone P. J., McCuen B. W., de Juan E., Machemer R. (1994). The efficacy of silicone oil for complicated retinal detachments in the pediatric population. *Archives of Ophthalmology*.

[24] Kuwabara R., Minamoto A., Yamane K., Kajikawa T., Mizote H., Mishima H. K. (2003). Retinal detachment in the mentally retarded. *Japanese Journal of Ophthalmology*.

[25] Bull M. J. (2011). Clinical report—health supervision for children with down syndrome. *Pediatrics*.

